# Bioaerosol Inactivation by a Cold Plasma Ionizer Coupled with an Electrostatic Precipitator

**DOI:** 10.3390/microorganisms12091923

**Published:** 2024-09-21

**Authors:** Samuel Wei Yang Lim, Sian Yang Ow, Laura Sutarlie, Yeong Yuh Lee, Ady Suwardi, Chee Kiang Ivan Tan, Wun Chet Davy Cheong, Xian Jun Loh, Xiaodi Su

**Affiliations:** Institute of Materials Research and Engineering (IMRE), Agency for Science, Technology and Research (A*STAR), 2 Fusionopolis Way, Innovis, #08-03, Singapore 138634, Singapore; samuel_lim@imre.a-star.edu.sg (S.W.Y.L.); owsy@imre.a-star.edu.sg (S.Y.O.); leeyy@imre.a-star.edu.sg (Y.Y.L.); adysuwardi@imre.a-star.edu.sg (A.S.); ivan_tan@imre.a-star.edu.sg (C.K.I.T.); davy_cheong@imre.a-star.edu.sg (W.C.D.C.); lohxj@imre.a-star.edu.sg (X.J.L.)

**Keywords:** cold plasma ionizer, non-thermal plasma, electrostatic precipitator, porcine respiratory coronavirus, *Escherichia coli*, bioaerosols, inactivation

## Abstract

Despite best efforts in air purification, airborne infectious diseases will continue to spread due to the continuous emission of bioaerosols by the host/infected person. Hence, a shift in focus from air purification to bioaerosol inactivation is urgently needed. To explore the potential of the cold plasma technology for preventing rapid spread of airborne infectious diseases, we studied a cold plasma ionizer (CPI) device and an electrostatic precipitator (ESP)-coupled CPI (CPI-ESP) device for the inactivation and cleaning of surface-spread microorganisms and bioaerosols, using porcine respiratory coronavirus (PRCV), *Escherichia coli* (*E. coli*), and aerosolized *E. coli* as representatives. We firstly demonstrated that CPI coupled with ESP is an effective technology for inactivating virus and bacteria spread on surfaces in an in-house test chamber. We then demonstrated the efficacy of CPI-coupled ESP for the inactivation of aerosolized *E. coli* in the same chamber. Furthermore, we have demonstrated the efficiency of a CPI-ESP coupled device for the inactivation of naturally occurring airborne microbials in a few indoor settings (i.e., a living room, a discussion room, a schoolroom, and an office) to determine the treatment duration- and human activity-dependent efficacy. To understand the disinfection mechanism, we conducted a fluorescence microscopy study to reveal different degrees of *E. coli* bacteria cell membrane damage under CPI treatment.

## 1. Introduction

Bioaerosol and surface transmission are the most common routes to spread infectious respiratory diseases, for instance, the SARS-CoV-2 virus [1,2,3]. Reducing the spread of such infectious diseases can be achieved by either reducing human exposure to bioaerosols (e.g., wearing surgical masks) or reducing the quantity of bioaerosols via inactivation/disinfection techniques.

For indoor spaces, bioaerosols can be removed by using high-efficiency particulate air filtration (HEPA filters) and electrostatic precipitators (ESPs) to adsorb bioaerosols, including mold spores, bacteria, pollen, viral particles, etc. [4,5,6,7]. HEPA filters remove particles from air that are forced through, but microorganisms are known to survive on a HEPA filter [8]. On the other hand, ESP removes particles from a gas stream by using electrical energy to confer a positive or negative charge to aerial particulates [9]. The charged particles are then attracted to collector plates carrying the opposite charge. ESP is mainly used to remove particulate matter (PM) in coal-fired power plants [10,11]. It is also possible to use an ESP for antibacterial/antiviral purposes due to the generation of additional electric fields, ions, and/or reactive species [12,13,14].

In addition to PM and bioaerosol trapping by HEPA and ESP, UV-C radiation, chemical sprays, and ionization of the air are popular approaches for bioaerosol decontamination [15,16,17]. Each of these technologies have their own drawbacks and application limitations. For example, direct human exposure to UV radiation is hazardous and can cause cancer and blindness. The efficiency of chemical disinfectant sprays depends on many factors, such as the concentration of the chemicals, reaction time, and presence of other organic matter that may react with the chemicals [18].

An emerging technology for the inactivation or disinfection of microorganisms is cold plasma or non-thermal plasma. Cold plasma or non-thermal plasma can be generated by the application of an electric or electromagnetic field to a gas, via various means, i.e., dielectric barrier discharge (DBD), atmospheric pressure plasma jet (APPJ), plasma needle, and plasma pencils [19,20]. The partially ionized gas is typically a mixture of excited atoms and molecules, electrons, ultraviolet photons, ions, and various other reactive species, such as singlet oxygen, ozone, hydroxyl radicals, and nitrogen oxides. The various oxygen radical species (atomic oxygen, hydroxide radical, superoxide radical, HO_2_, H_2_O_2_) and nitrogen radicals (atomic nitrogen, nitrogen oxides, and peroxynitrite) are known to have an excellent bactericidal effect on microbes [21], by compromising cell membranes and damaging proteins and nucleic acids [19,22]. Cold plasma treatment has been demonstrated to be effective against bacteria [19,23,24], bacterial endospores [25,26], microbial toxins [25], fungi [27], and viruses [20,27,28,29] in various applications, including food production [25,30,31], wound healing [32], and medical device sterilization [33,34]. Cold plasma technology can also be used to disinfect N95 masks to allow for safe re-use of the mask [35], and reducing the environmental impact from widespread use of masks.

When faced with disinfection techniques, microorganisms tend to develop a resistance to the particular techniques due to selective pressures [30]. In the case of cold plasma, an upregulation of catalase or redox-active chemical production in bacteria could cause the bacteria to develop a tolerance towards cold plasma exposure [19,36]. As such, coupling an electrostatic precipitator with cold plasma treatment could be more effective in preventing the development of resistance towards airborne inactivation/disinfection and improve the overall inactivation efficacy.

To explore the potential of the cold plasma technology for preventing rapid spread of airborne infectious diseases and prove the hypothesis that an ESP can promote the cold plasma treatment, in this study, we systematically studied a dielectric barrier discharge (DBD)-type cold plasma ionizer (CPI) for its efficiency to inactivate bacteria (using *E. coli* as the model organism) and a virus (using porcine respiratory coronavirus (PRCV) as a surrogate for SARS-CoV-2), with and without coupling to an electrostatic precipitator (ESP). We firstly studied the capability of a CPI and a CPI coupled with an ESP (CPI-ESP) to inactivate *E. coli* or PRCV on surfaces (without aerosolization) and then for *E. coli* aerosolized in air using a nebulizer. A testing chamber was built to host the *E. coli* samples, the CPI-ESP coupled apparatus, PM (particulate matter) meters, a particle analyzer, an ozone meter, and the nebulizer. For air inactivation studies, the chamber is further equipped with a liquid impinger air sampler to collect the aerosolized *E. coli* before and after CPI or CPI-ESP treatment. Following the study in the test chamber, we demonstrate the efficiency for general bacteria inactivation in various air-conditioned indoor settings, i.e., an office room, a living room, and a discussion room, with which we have demonstrated the treatment duration- and volume space-dependent inactivation efficacy of commercial CPI-ESP equipment. To understand the inactivation mechanism and address the safety concerns, we monitored the ozone level in the chamber and in a discussion room (one of the air-conditioned indoor settings studied). With this study, we demonstrated that the CPI-ESP is an effective and safe tool for bioaerosol inactivation.

## 2. Materials and Methods

### 2.1. Biological Agents and Cultivation Processes

Porcine respiratory coronavirus (PRCV) was purchased from ATCC (Manassas, VA, USA) (CRL-2384). The virus was propagated in ST cells (ATCC CRL-1746) grown on Minimum Essential Medium (MEM) containing 2 mM glutamine and Earle’s balanced salts (Cytiva, Marlborough, UK), with the addition of 1 mM sodium pyruvate (Gibco, Waltham, MA, USA), 1× MEM Non-essential Amino Acid Solution (Sigma, St. Louis, MO, USA), and 2% fetal bovine serum (Gibco, Waltham, MA, USA) according to ATCC’s growth conditions. The virus was separated from host cells by centrifugation after observed cytopathic effects and stored in stock solution aliquots under −80 °C. The concentration of PRCV in the stock solution aliquots was determined by a Median Tissue Culture Infectious Dose (TCID_50_) assay to be 6.95 × 10^8^ TCID_50_/mL, taking an average of the estimated viral concentration as calculated by the Reed–Muench method and the improved Kärber method. PRCV was stored in the ATCC-recommended viral media, which consist of MEM supplemented with ~1 mM sodium pyruvate and 1× non-essential amino acids.

*Escherichia coli* (*E. coli*) strain ATCC 25922 was purchased from ATCC and grown on nutrient agar (Oxoid, Waltham, MA, USA) in an incubator for 24 h at 37 °C. To prepare a working culture, freshly cultured colonies of *E. coli* were suspended in sterile 0.9% NaCl or 1× PBS Phosphate-Buffered Saline (PBS). The suspensions were diluted to an optical density at 600 nm (OD_600_) value of 0.25, measured using Shimadzu™ Spectrophotometer Biospec-Mini [37] (Shimadzu Coporation, Tokyo, Japan), which corresponds to 1–2 × 10^8^ CFU/mL. A plate count was performed post experiment to determine the actual *E. coli* concentration.

### 2.2. Apparatus and Testing Chamber

**Figure 1:**
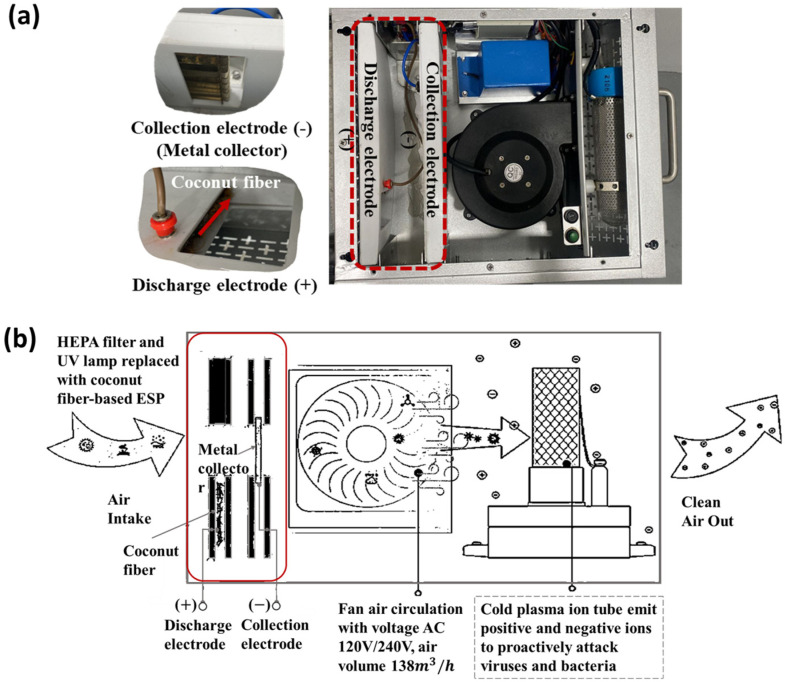
(**a**) In-house-built CPI-ESP from partially modified EddaAir cold plasma device. (**b**) Schematic drawing of components inside CPI-ESP.

An in-house-built CPI coupled with an ESP was prepared by the modification of an air disinfection system procured from EddaAir (Shenzhen, China). The CPI tube (in the EddaAir device) is 180 mm long with power specifications of 12 VDC and 50 W. The air volume sampling rate is 138 m^3^/h. For the purpose of this study, we substituted the HEPA filters and UV lamp with an ESP component comprising a discharge electrode and metal collector according to the work by Suwardi et al. [38] as shown in Figure 1. During this study, the power input and fan speed were not changed. Hence, the plasma-induced ROS and RNS generation profiles and air flow rate were presumed to be fixed based on the specifications of the EddaAir product.

**Figure 2:**
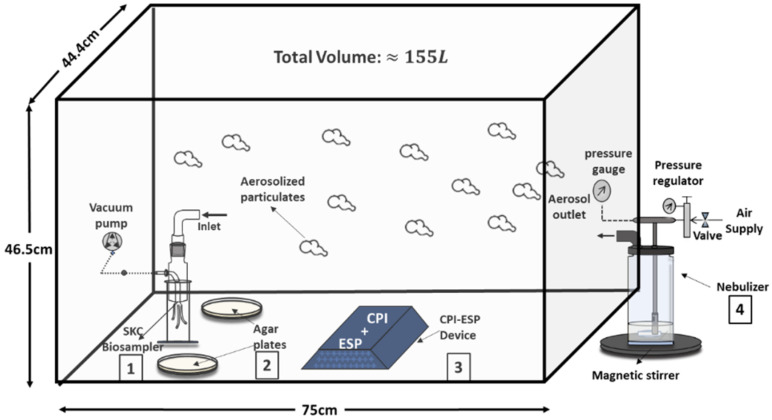
Schematic drawing of bioaerosol testing chamber and apparatus components. (1) SKC BioSampler for active sampling. (2) Agar plates for passive sampling. (3) CPI-ESP device. (4) Nebulizer.

This modified CPI-ESP device was used for all experiments related to the lab strain virus and bacteria that were performed in an in-house-built closed acrylic chamber (75 × 44 × 47 cm with a total volume of 155 L) illustrated in Figure 2. This chamber hosts four main components, namely an active sampler (SKC BioSampler, Eighty Four, PA, USA), a passive sampler (agar plates), a CPI-ESP device, and a nebulizer. The chamber is placed in a sterile biosafety cabinet for all the experiments. The positions of the air samplers (SKC BioSampler and agar plates) within the test chamber are determined based on methodology discussed in Section 2.5.2. The CPI and ESP are modular and can be operated separately. The ESP component was turned off when the CPI effect was studied. Both the CPI and ESP components were turned on when studying the coupled effects of CPI and ESP.

**Figure 3:**
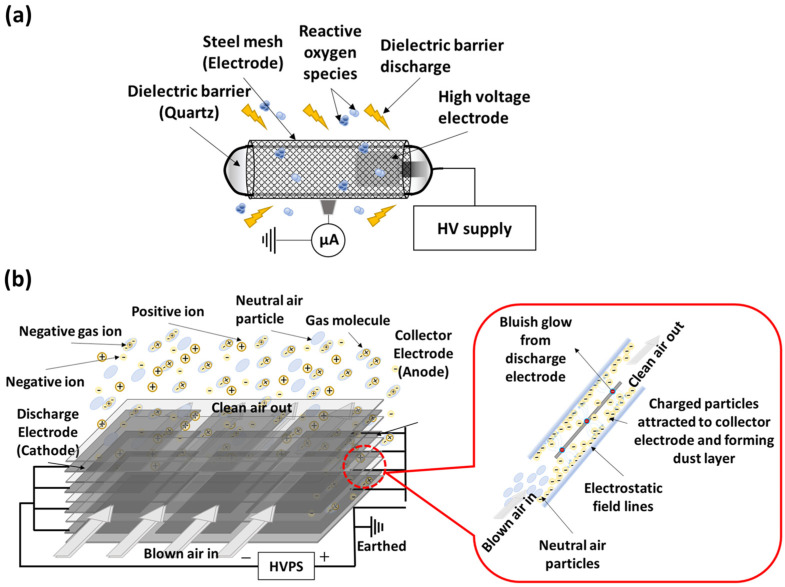
Schematics of (**a**) dielectric barrier discharge (DBD)-based CPI and (**b**) ESP.

A commercial air disinfection system, Airdome™70 (Plasma Science Pte. Ltd., Singapore), containing similar coupling of a CPI with an ESP was deployed for the bioaerosol inactivation study in various indoor settings (for experimental details, see Section 2.7). This commercial system integrated a CPI tube with an ESP apparatus that consists of a paired collection and discharge electrode plates. The operational principles of the CPI and ESP are illustrated in Figure 3. The CPI generates reactive oxygen and/or nitrogen species responsible for virus inactivation through effects on capsid proteins and/or nucleic acids (Figure 3a) [29]. ESP is a platform that can attract microbial particles by electrostatic interactions [39]. The ESP used in this study is a two-stage precipitator, where the charging field and the collecting field are independent of each other (Figure 3b). Corona discharge occurring in the ESP may contribute to the total amount of disinfection species emitted by the combined CPI-ESP device. To operate this device, the specified clean air delivery rate (CADR) was set at 400 m^3^/h. Ozone concentration was monitored using an Indoor Air Quality (IAQ) certified tool (model: Aeroqual Series 500, Auckland, New Zealand).

### 2.3. Inactivation of Porcine Respiratory Coronavirus Droplets on Surfaces

Porcine respiratory coronavirus (PRCV) spread on glass coverslips and/or *E. coli* agar plates was placed in the chamber close to the CPI-ESP device. Glass coverslips were sterilized via 10 min UV irradiation on both sides in a biosafety cabinet (BSC) hood using the UV lamp. In the test chamber, a virus solution (10 µL) was spread as ten microdroplets on a sterile glass coverslip (two duplicate coverslips in each experiment; repeated twice). The CPI-ESP device in the chamber was then turned on for different durations of exposure time (from 5 to 60 min). After the desired exposure time, the virus samples were then taken out from the chamber and recovered by the addition of 1 mL of viral media to the glass coverslip and the virus was resuspended via pipetting. The concentration of live virus in the collected viral media from each sample was analyzed via a TCID_50_ assay. The same amount of virus solution spread on a glass slide but not exposed to CPI or CPI-ESP was used as a negative control. The live virus titer in the control and after inactivation treatment was calculated after 5 days of observation. Where no cytopathic cells were observed in any of the treated wells, we assumed the final viral titer as 6.31 TCID_50_/mL, which is the estimated limit of detection of such assays [39]. The inactivation efficiency was calculated as the percentage reduction of the live virus after exposure to CPI or CPI-ESP treatment, relative to the control by Formula (1):

(1) $$Percentage\;reduction\;\left(\%\right)=\frac{{{TCID}_{50}}^{Control}-{{TCID}_{50}}^{CPI\;(CPI-ESP)}}{{{TCID}_{50}}^{Control}}\times 100$$

### 2.4. Inactivation of E. coli on Surfaces

In the testing chamber, 100 µL of 10^4^ to 10^3^ CFU/mL *E. coli* suspended in sterile 0.9% NaCl was spread on agar plates using an L-spreader (2 duplicates) and placed at ~20 cm away from the CPI (or CPI-ESP) inside the chamber. The CPI (or CPI-ESP) apparatus placed in the middle inside the test chamber was turned on for various exposure times (5 to 60 min). After exposure, the agar plates were removed for incubation at 37 °C in an incubator overnight. The same batch of *E. coli* spread on separate agar plates not exposed to CPI or CPI-ESP were used as a negative control. The number of bacteria colonies on the agar plates was counted after overnight incubation. This experiment was repeated at least twice for each time point. The inactivation efficiency was calculated as the percentage reduction of colony forming units (CFUs) left on the plate after exposure to treatment relative to the control by the following Formula (2):

(2) $$Percentage\;reduction\;\left(\%\right)=\frac{{CFU}^{Control}-{CFU}^{CPI\;(CPI-ESP)}}{{CFU}^{Control}}\times 100$$

### 2.5. Aerosolization and Inactivation of E. coli in a Bioaerosol Chamber

In this section, the methods are described for three related processes, i.e., the aerosolization of *E. coli*, air sampling, and inactivation study.

#### 2.5.1. Aerosolization of *E. coli*

Firstly, a Collison Nebulizer (CH Technologies, Westwood, NJ, USA) was used to generate bioaerosols from the *E. coli* solution (75 mL of 10^6^ or 10^5^ CFU/mL in sterile 1× PBS). The nebulizer was operated at an air flow rate of 2 to 4 L/minute (LPM). Particulate sensors Sensirion SPS30 (Sensirion AG, Chicago, IL, USA) were placed in the chamber to characterize the generated bioaerosol particle concentration and size profile, with real-time data sent wirelessly to a computer. The sensors were calibrated using TSI DustTrak^TM^ DRX Aerosol Monitor 8533 (TSI Incorporated, Shoreview, MN, USA) as a reference. The particle concentration and size profiles of aerosolized *E. coli* generated by the nebulizer at various air flow rates are shown in Figure S1.

#### 2.5.2. Air Sampling and Samplers’ Position

The distribution of the *E. coli* aerosols across the test chamber was characterized by passive sampling with agar plates placed on various spots of the floor in the chamber (Figure S2a) over 10 min of simultaneous aerosolization by the nebulizer and the passive sampling. Based on the aerosol distribution results (Figure S2b–e), the optimal positioning of the SKC BioSampler for the bioaerosol inactivation experiment was determined at position A2 facing the nebulizer.

#### 2.5.3. Inactivation of Aerosolized *E. coli*

In the bioaerosol inactivation experiment, the nebulizer was turned on for a total of 20 min, while at the 5 min time point, the SKC BioSampler was turned on for 30 min. The SKC BioSampler was filled with 20 mL of sterile 1× PBS for the collection of the *E. coli* bioaerosols and operated with a pump at a flow rate of 12.5 L/minute. Two nutrient agar plates were placed on each side of the SKC BioSampler and were equally spaced apart to account for the settlement of bioaerosols (as a passive sampler). The CPI-ESP device was started either prior to the nebulizer, allowing the test chamber to be saturated with CPI-ESP emissions, or at the same time as the nebulizer was turned on. To determine the inactivation efficacy, the *E. coli* collected by the two passive agar samplers and the SKC BioSampler were recorded for experiments with and without the operation of the CPI-ESP device, when nebulization conditions and air sampler conditions (e.g., location, etc.) were fixed. Equation (2) above was then applied to calculate the percentage reduction, where the control counts are from no CPI-ESP operation. The passive agar samplers were incubated for 24 h at 37 °C, before the number of bacteria colonies on each plate was counted. The recovered *E. coli* by the SKC BioSampler were determined by spreading 100 µL of the collection media onto nutrient agar plates (in duplicates) for culturing under the same conditions (24 h incubation at 37 °C). The colony count from these plates was then back-calculated, i.e., multiplied by 200 to acquire the total CFU of *E. coli* recovered by the liquid air sampler.

### 2.6. Fluorescence Microscopy Studies of E. coli Inactivation

Fluorescence microscopy studies were conducted using a Leica DM5000B microscope (Leica, Wetzlar, Germany) and an improved Neubauer cell counting slide using 10× magnification. Before viewing under the microscope, 10 µL of a sample was mixed with 10 µL of Baclight^TM^ viability assay stain and incubated for 5 min. Five separate images were obtained by using the GFP (excitation of 430–510 nm, emission of 475–575 nm) filter and N21 (excitation of 515–560 nm, emission of 590 nm) filter. The stain is a mixture of two DNA-binding fluorescent dyes: SYTO9 and propidium iodide (PI). The former is used to identify all bacteria and the latter to identify bacteria with a compromised cell membrane (i.e., dead cells) [40,41]. Total bacteria (live and dead) were estimated by counting the number of fluorescent particles observed in the GFP filter, which are SYTO9 dye-stained cells. Then, the dead bacteria were estimated by counting the number of fluorescent particles under the N21 filter, which are PI-stained dead cells.

### 2.7. Bioaerosol Inactivation Studies in Indoor Settings

The bioaerosol inactivation studies by using CPI-ESP in indoor settings were performed in indoor air-conditioned public spaces with commercial CPI-ESP equipment (Airdome™70, Plasma Science Pte. Ltd., Singapore). This commercial CPI-ESP equipment has a clean air delivery rate (CADR) of up to 400 m^3^/h that mimics the open air factor (OAF) condition [42,43] by generating reactive species during air ionization and delivering revitalized clean air.

With concerns related to ozone generation by CPI, we firstly performed ozone monitoring measurements with and without the commercial CPI-ESP equipment in a meeting room of a 32.5 m^3^ volume space. Each monitoring test runs for a duration of 8 h, referenced to the guidelines by National Environmental Agency (NEA), Singapore. We then performed the bioaerosol inactivation study by CPI-ESP in three empty indoor settings (no human activities), i.e., a living room and a discussion room of the same volume space of 54 m^3^, as well as an office of 375 m^3^. The commercial CPI-ESP coupled air purifier was operated for either 1 or 24 h; after that, a nutrient agar-based FKC-III microbial air sampler was used to sample the air. This FKC-III air sampler was operated for 20 min with a flow rate of 100 L/minute before and after CPI-ESP treatment. The agar plates were then incubated at 37 °C for 5 days before the various bacteria and fungi colonies were counted. The Aerotrak™ particle counter was also operated with a flow rate of 2.83 L/minute for the same duration of 20 min, to count 1 µm particulate matter.

## 3. Results

### 3.1. Inactivation of Porcine Coronavirus and Escherichia coli on Surfaces

**Figure 4:**
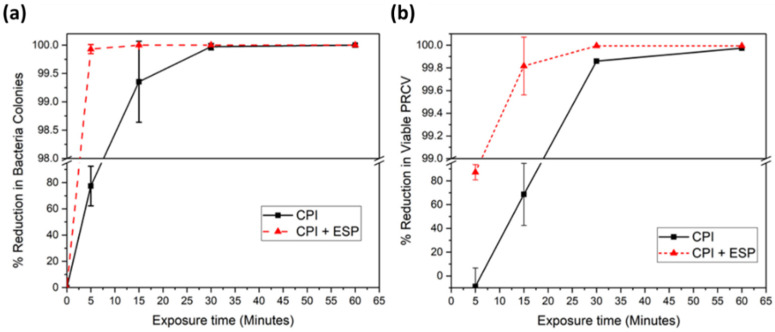
Percentage reductions of (**a**) *E. coli* and (**b**) PRCV in the testing chamber over time. The standard deviations at 5 min and 15 min are 6.43% and 0.25%, respectively. The standard deviations beyond 30 min remained as zero from no viable counts.

We first studied the capability of CPI and the combination of CPI-ESP to inactivate PRCV and *E. coli* on surfaces. The inactivation outcomes by the CPI device and the integrated CPI-ESP device were plotted as percentages of reduction in the number of microbes as a function of treatment time (Figure 4).

For *E. coli,* the integrated CPI-ESP device was able to achieve 99.9% or a 3-log reduction in the bacteria within 5 min of exposure, whereas by CPI alone, 30 min was needed to achieve the same degree of reduction shown in Figure 4a. For PRCV, a similar degree of inactivation (99.8% or 3-log reduction) was achieved in 15 min by the CPI-ESP device, whereas the CPI alone only achieved 60% (<1-log reduction) for the same 15 min treatment shown in Figure 4b. At 30 min of treatment, 99.99% (4-log reduction) of PRCV was achieved by the CPI-ESP, whereas that by CPI was only a 3-log reduction. For both *E. coli* and PRCV, the coupled CPI-ESP device shows higher inactivation efficiency than CPI alone. While the ESP component is expected to be more effective against bioaerosols as a precipitator for bioaerosols, the higher inactivation efficiency shown by the combined CPI-ESP device for the *E. coli* and PRCV on surfaces could be due to the generation of additional electric fields, ions, or reactive species from the ESP device [12,13,14,39] that attacked the bacteria and virus on the surface.

Additionally, the data in Figure 4 present an interesting note that *E. coli* is more susceptible to CPI (and CPI-ESP) inactivation than PRCV. A 3-log reduction in *E. coli* was achieved within 5 min of exposure to CPI and CPI-ESP, compared to 15 min for PRCV to achieve the same degree of inactivation. In an earlier study of the UV disinfection of *E. coli* and an MS2 bacteriophage (an icosahedral, positive-sense single-stranded RNA virus), the higher susceptibility of *E. coli* to UV disinfection than MS2 was observed as well [34]. The relatively strong resilience of the virus compared to bacteria has been observed in other disinfection methods by UV of other biocides in that structural damage to viral capsids and even to DNA polymerases may not always result in the loss of infectivity of the virus [44].

### 3.2. Inactivation Study of E. coli Bioaerosol

**Figure 5:**
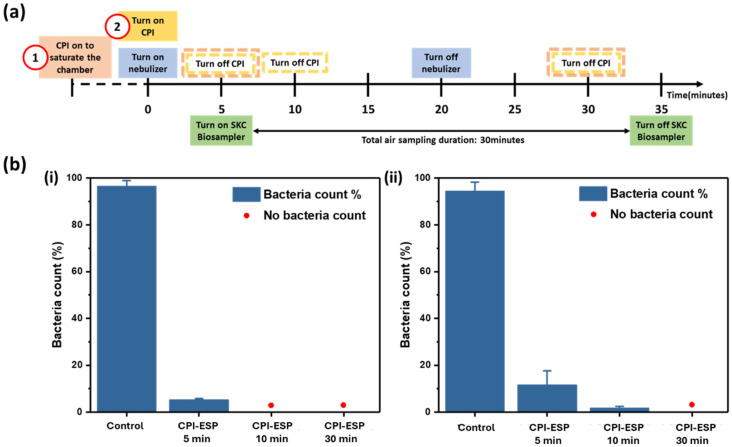
(**a**) Timelines illustrating the operational sequence of the nebulizer, SKC BioSampler, and CPI device for the bioaerosol inactivation study. The CPI operation was in two arrangements: 1 and 2. (**b**) The inactivation of aerosolized *E. coli* with CPI operational timeline arrangement 2, using (i) active sampling by SKC BioSampler and (ii) passive sampling on an agar plate.

Upon confirming the superior performance of coupled CPI-ESP relative to CPI alone using surface-spread microorganisms (PRCV and *E. coli*), here, we study the CPI-ESP efficacy in inactivating aerosolized *E. coli*. As mentioned in Experimental Section 2.5.3, we have designed the experiments with two different timelines of CPI-ESP operation, relative to the operation of the nebulizer, as shown in Figure 5a. The original *E. coli* count without CPI-ESP treatment (control) was ~40,000 CFU and ~3000 CFU from the SKC sampler and the passive agar samplers, respectively. In CPI-ESP operational timeline arrangement 1, where the chamber was filled with ions prior to *E. coli* aerosol generation, with as short as 5 min of CPI-ESP treatment, no bacteria colonies were grown from SKC collection liquid, nor on the passive sampling agar plates at the end of 30 min of sampling (Figure S3). Hence, at least 4-log and 3-log reduction in viable *E. coli* were measured by the SKC and agar samplers, respectively. In the next experiment and to verify the deactivation and killing effect of cold plasma ions at a lower concentration, when the CPI was turned on later, together with the nebulizer (CPI operational timeline arrangement 2), at the 5 min treatment, decreased inactivation efficiency was observed as expected, i.e., 5.1% and 13.8% of viable *E. coli* remain detectable (correlated to >1-log and <1-log reduction), respectively, by the two sampling methods as shown in Figure 5b (the respective bacteria count results are in Figure S4). A prolonged 10 min is needed for treatment under timeline arrangement 2, in order to achieve the same degree of reduction as that by 5 min of treatment under arrangement 1.

In comparison to the inactivation of *E. coli* on surfaces, where only 3-log reduction was achieved after 30 min of exposure to CPI, the *E. coli* bioaerosol inactivation reached 4-log reduction at a faster time at 10 min of CPI exposure. Quicker inactivation by the CPI treatment to bioaerosols could be due to the significantly higher contact area of aerosolized particles to CPI disinfection species, while the bacteria on surfaces are only partially exposed to the atmospheric CPI disinfection species.

### 3.3. Fluorescence Microscopy Study of E. coli Death in Bioaerosols

**Figure 6:**
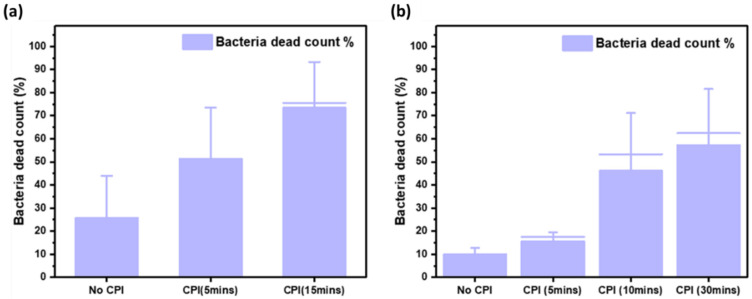
The average percentage of dead bacteria from fluorescence microscopy counting. Error bars represent the standard deviation. (**a**) CPI treatment starting at the beginning of the experiment in CPI as-saturated test chamber. (**b**) CPI treatment starting at the beginning of the experiment.

To uncover the inactivation mechanism and effect of atmospheric CPI disinfection species on the *E. coli* bioaerosols, the fluorescence microscopy characterization of the liquid collected from the SKC BioSampler with and without CPI-ESP treatment was performed using SYTO9 and PI dyes. Figure 6 shows the ratio of dead cells to total cells (live and dead) with and without CPI-ESP treatment following operational timeline arrangement 1 (saturated CPI, Figure 6a) and arrangement 2 (un-saturated CPI, Figure 6b).

In the absence of CPI, about 10% to 27% of the collected bacteria are dead as confirmed by PI fluorescence, indicating a damaged membrane (Figure S5). When the CPI-ESP device is operated, the proportion of the dead cells increased in a time-dependent manner. There was a presence of “live” cells that have intact membranes as confirmed by the fluorescence microscopy with the SYTO9/PI staining (Figures S6 and S7), despite the absence of bacteria colony forming units from plate counts of the same SKC collection liquid. This indicates that the bacteria may have entered a “viable but non-culturable” state or may have otherwise been rendered non-viable without membrane perforation from the CPI-ESP treatment. Both the states are likely a result of damage to various cell surface components. Additionally, the presence of more dead bacteria with membrane perforation at a higher exposure time suggests that the cumulative damage from longer CPI exposure eventually results in membrane integrity failure and cell death. The presence of fluorescent bacteria in the fluorescence microscopy studies also shows that CPI exposure under these conditions does not significantly break up DNA, as both SYTO9 and PI are DNA intercalating dyes.

When comparing between the two CPI-ESP operational arrangements, within 5 min, there was a much larger proportion of dead cells (52%) for the saturated CPI operation as compared to non-saturated operation (15%). This indicates that the viable bacteria concentration decreased with longer CPI saturation conditions. Studies in a review by Zhang et al. on bactericidal properties of cold plasma have explained the bacteria inhibition mechanisms [45]. The actions of ROS and RNS species generated by CPI include the oxidation and perforation of the cell membrane, degradation and modification of proteins, and modification and chain-breaking of nucleic acid molecules. Since we used *E. coli*, a Gram-negative bacillus with a thin peptidoglycan cell wall, in this study, plasma-induced reactive species should mainly damage the cells via membrane peroxidation, thus inducing leakage of intracellular material [46].

### 3.4. Bioaerosol Inactivation in Indoor Room Settings

After the inactivation study using the partially self-constructed CPI-ESP device to treat experimentally aerosolized *E. coli* in the test chamber, we now study and demonstrate the disinfection efficacy of a commercial CPI-ESP coupled air purifier for naturally occurring airborne microorganisms in a few indoor room settings, i.e., a living room, a discussion room, and an office, of varied space volume. Prior to the bioaerosol inactivation study in indoor settings, we conducted ozone monitoring over a period of 8 h in 10 min intervals with and without the commercial CPI-ESP setup to determine if the ozone level is within the safe limit based on safety guidelines by NEA, Singapore. The ozone monitoring results show that the operation of the commercial CPI-ESP setup did not lead to a significant increase in the ozone level, which is within the safety limits of 0.05 ppm (Figure S8).

**Figure 7:**
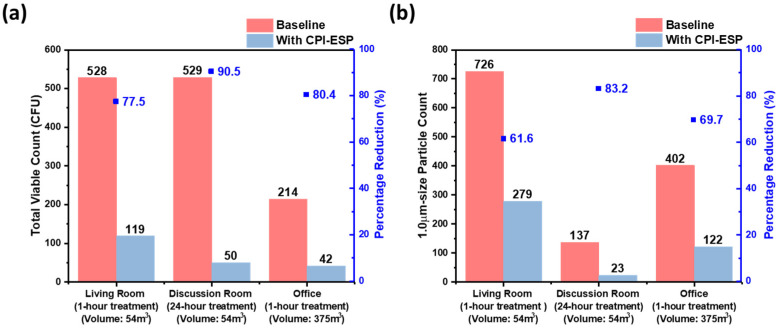
(**a**) Total viable count (N = 1) and (**b**) 1 μm size particle count results (N = 1) from a living room and a discussion room of a similar room volume for 1 h of treatment and 24 h of treatment and an office with a larger volume for 1 h of treatment by the commercial CPI-ESP equipment.

Upon confirmation that the ozone level was within safe limits, we determined the treatment duration-dependent inactivation efficacy in two unmanned indoor rooms of approximately a 54 m^3^ volume, i.e., a living room and a discussion room. Results (Figure 7a) show that 528 CFU and 529 CFU of bacteria were collected prior to the CPI-ESP treatment from the living room and discussion room, respectively, while only 119 CFU and 50 CFU of bacteria were collected from the living room with 1 h of CPI-ESP treatment and the discussion room with 24 h of CPI-ESP treatment, respectively. The inactivation efficacy from 24 h of treatment (90.5% and ~ 1-log reduction) is higher than the 1 h of treatment (77.5%, <1-log reduction), as expected. We also measured the indoor air PM concentration (Figure 7b). An average of 726 and 137 particle counts in 20 min sampling time were observed in the absence of CPI-ESP treatment, while only 279 and 23 particle counts were observed with CPI-ESP treatment from the living room and discussion room, respectively. A higher air cleaning efficacy (83.2%) is observed from the particle count results after 24 h of treatment, which is better than 1 h of treatment (61.6%). This demonstrates the time-dependent effectiveness of CPI-ESP systems against naturally occurring airborne microorganisms and airborne particulates.

Next, to study the effectiveness of CPI-ESP bioaerosol inactivation in indoor settings of different space volumes, the commercial CPI-ESP equipment was placed in an office of a larger space volume of 375 m^3^ and operated for 1 h. The result is compared with those for the smaller living room (54 m^3^) with the same 1 h treatment. As shown in Figure 7a, the initial viable count in the larger office is 214 CFU. After 1 h of treatment, it dropped to 42 CFU, i.e., 80.4% (<1-log) reduction. The reduction rate is similar to that for the smaller living room (77.5% or <1-log reduction). For the indoor air PM concentration (Figure 7b), a 69.7% reduction is obtained in the office, which is also similar to that in the living room (61.6%). These results show that even in a large-space-volume indoor setting (the office is about seven times the living room), airborne microbial inactivation and air cleaning efficacy remain significant or as effective as in a small room. This can be attributable to the large spatial coverage of the active species from the CPI-ESP device.

It is worthwhile to note that the exact log reduction obtained here for indoor settings using the commercial machine is different from that of the inactivation studies in the test chamber using the partially self-constructed CPI-ESP device. This is because of the differential microorganisms involved (naturally occurring environmental microorganisms vs. *E. coli*) and differential environmental conditions (humidity and temperature, etc.). In addition, the two devices have intrinsic differences in their physical and electrical specifications, e.g., CPI tubes, design of the electrostatic precipitator (Figure 1a and Figure 3b), power specifications, and clean air delivery rate (CADR) (Table S1). Nevertheless, our studies have confirmed the effectiveness of a CPI-ESP or CPI-ESP coupled air purifier for the inactivation of microorganisms in various settings, including real-world scenarios.

## 4. Conclusions

We have investigated the efficiency of a CPI device and an ESP-coupled CPI device (CPI-ESP) for bioaerosol inactivation. Through controlled experiments in a bioaerosol chamber, we have demonstrated that the CPI-ESP combination is more effective in the inactivation of the virus and bacteria (both aerosolized in air and spread on surfaces) than CPI alone; and the reduction in the aerosolized viable microorganism (*E. coli*) by the CPI-ESP device is faster than the reduction in the surface-spread microorganism. We have also exploited a commercial air purifier that consists of CPI and ESP to study the bioaerosol inactivation in indoor settings to demonstrate the ability of this machine in inactivating naturally occurring environmental microorganisms, in treatment time- and space volume-dependent manners. Using the fluorescence microscopic technique, we have revealed the inactivation mechanism that involves the perforation of the bacterial cell membrane. We believe that this work can enhance the understanding of the mechanism of bioaerosol inactivation by CPI-ESP and provide guidance for the future optimization and practical use of these combined technologies. Further studies can be performed to understand the effect of various environmental conditions (humidity, temperature, etc.) on the inactivation efficiency for a given CPI-ESP device, or the impacts of device specifications on inactivation efficiency.

## Data Availability

The original contributions presented in the study are included in the article/supplementary material, further inquiries can be directed to the corresponding authors.

## Supplementary Materials

The following supporting information can be downloaded at: https://www.mdpi.com/article/10.3390/microorganisms12091923/s1, Table S1: Specification of the CPI-ESP devices; Figure S1: Characterization of Aerosolized *E. coli*; Figure S2: Plate culture mapping of aerosolized *E. coli* in the chamber; Figures S3 and S4: Bacteria count of aerosolized *E. coli* collected by active sampler (SKC BioSampler) and passive sampler (Agar plate); Figures S5–S7: Fluorescence microscopy of *E. coli* collected by SKC BioSampler with CPI treatment duration of 0-, 5-, 15-min, respectively; Figure S8: Ozone data obtained in the meeting room without and with CPI-ESP treatment.
