# Supplementary material for: Bioaerosol Inactivation by a Cold Plasma Ionizer Coupled with an Electrostatic Precipitator
Source: Microorganisms. 2024 Sep 21;12(9):1923. doi: 10.3390/microorganisms12091923 (PMC11433785; doi:10.3390/microorganisms12091923)
Supplement: Supplementary file 1 [file microorganisms-12-01923-s001.zip › microorganisms-3147488-supplementary.pdf]

Supporting Information

Bioaerosol Inactivation by a Cold Plasma Ionizer Coupled with an Electrostatic Precipitator

Samuel Wei Yang Lim, Sian Yang Ow, Laura Sutarlie *, Yeong Yuh Lee, Ady Suwardi †, Chee Kiang Ivan Tan, Wun Chet Davy Cheong, Xian Jun Loh and Xiaodi Su *

Institute of Materials Research and Engineering (IMRE), Agency for Science, Technology and Research (A*STAR), 2 Fusionopolis Way, Innovis, #08-03, Singapore 138634, Singapore; samuel_lim@imre.a-star.edu.sg (S.W.Y.L.); owsy@imre.a-star.edu.sg (S.Y.O.); leeyy@imre.a-star.edu.sg (Y.Y.L.); adysuwardi@imre.a-star.edu.sg (A.S.); ivan_tan@imre.a-star.edu.sg (C.K.I.T.); davy_cheong@imre.a-star.edu.sg (W.C.D.C.); lohxj@imre.a-star.edu.sg (X.J.L.)

* Correspondence: laura-sutarlie@imre.a-star.edu.sg (L.S.); xd-su@imre.a-star.edu.sg (X.S.)

† Current address: Department of Electronic Engineering, The Chinese University of Hong Kong, New Territories, Hong Kong SAR 999077, China.

Table S1. Key specifications of the two devices

	**Modified EddaAir for in-chamber study**	**Airdome™70 for indoor settings study**
**Cold Plasma Ionization Tube**	**Dimensions:**<br>180 mm (Length) ×38 mm (Diameter)<br>**AC power supply (Pulsed):**<br>6 Watts, 220 VAC, 50Hz	**Dimensions:**<br>80 mm (Length) × 28 mm (Diameter)<br>**DC power supply:**<br>1 Watts, 5 VDC
**Electrostatic Precipitator**	Coco fibre as a discharge electrode and metal plate as a collection electrode (Figure 1)	Both discharge and collection electrodes are polymeric plates (Figure 3b)
**Power specifications of the System**	Rated Voltage: 24 VDC<br>Power: 50 W	Rated Voltage: 24 VDC<br>Power: 60 W
**Clean Air Delivery Rate (CADR)**	138 m^3/hour	400 m^3/hour

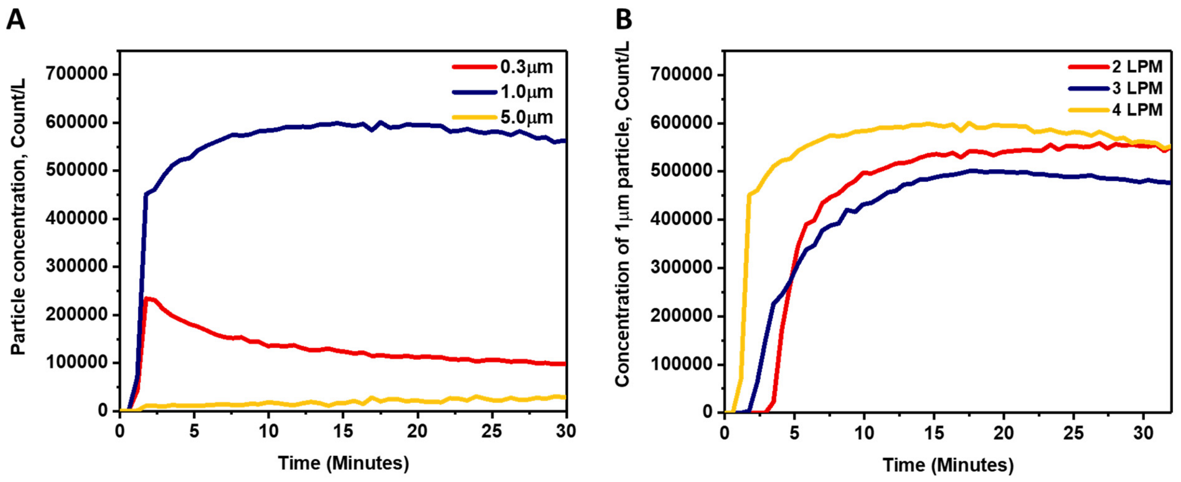

Figure S1. (**a**) Particle size profiles for aerosolized *E. coli* and (**b**) 1 µm aerosol concentration generated by the nebulizer at different air flow rates in liter per minute (LPM).

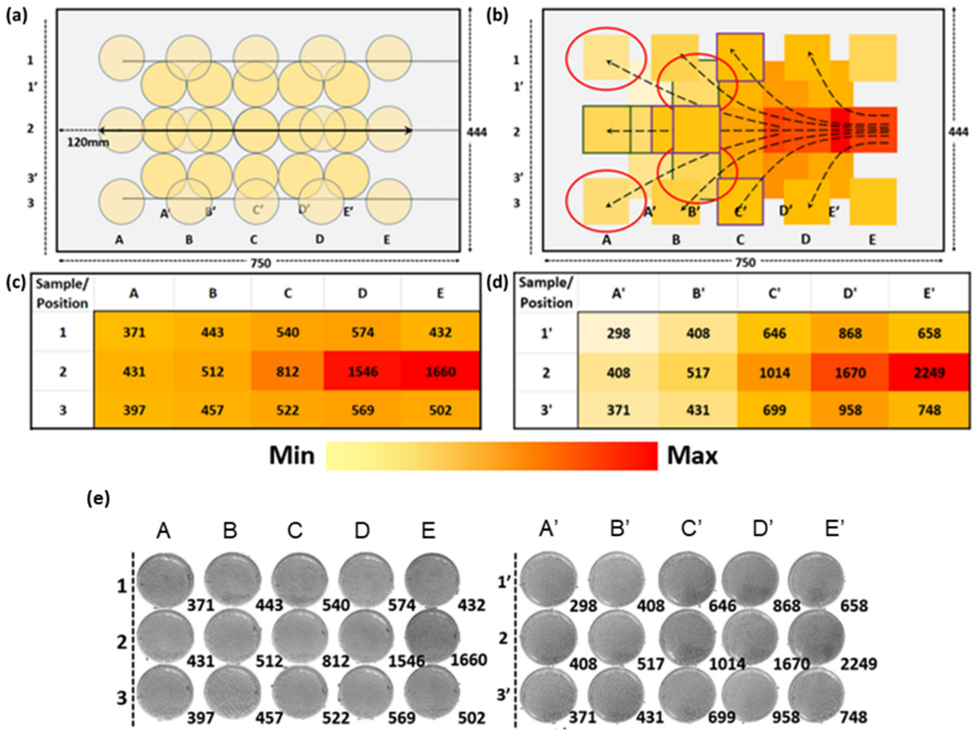

Figure S2. Mapping of aerosolized *E. coli* (from 10^5 CFU/mL solution in the nebulizer) in the aerosol test chamber using passive agar sampling. (**a**) Agar plates arrangement, (**b**) Heatmap derived from plate counts of the two sets agar plates ((**c**) and (**d**)). The numbers in (**c**) and (**d**) are the number of *E. coli* colonies obtained on the agar plates. **(e)** Plate count images of the aerosolized *E. coli* mapping in the testing chamber.

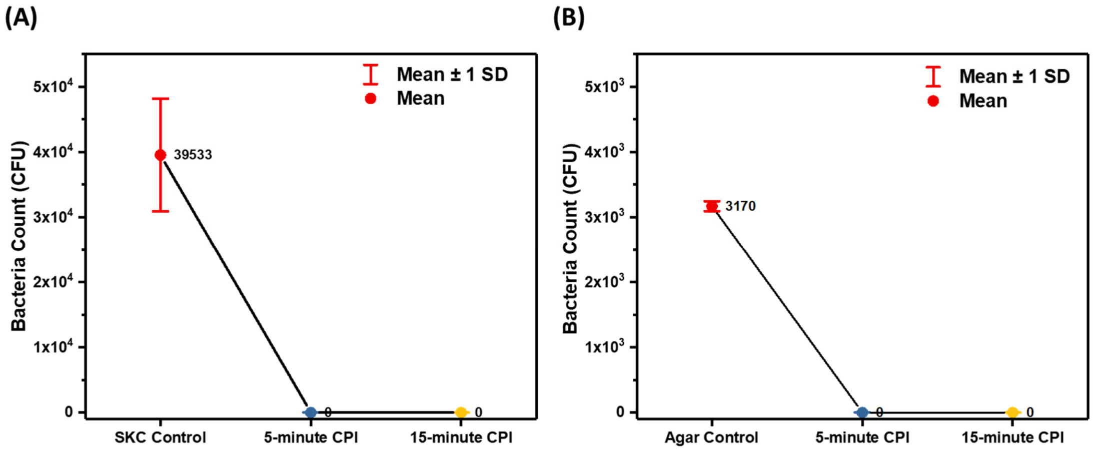

Figure S3. Bacteria count results from collected aerosolized *E. coli* (from 10^5 CFU/mL solution in the nebulizer) in the aerosol test chamber using SKC BioSampler and passive agar sampling after exposure duration to CPI based on operational timeline arrangement 1. (**a**) SKC BioSampler bacteria count results (**b**) Passive agar sampling bacteria count results.

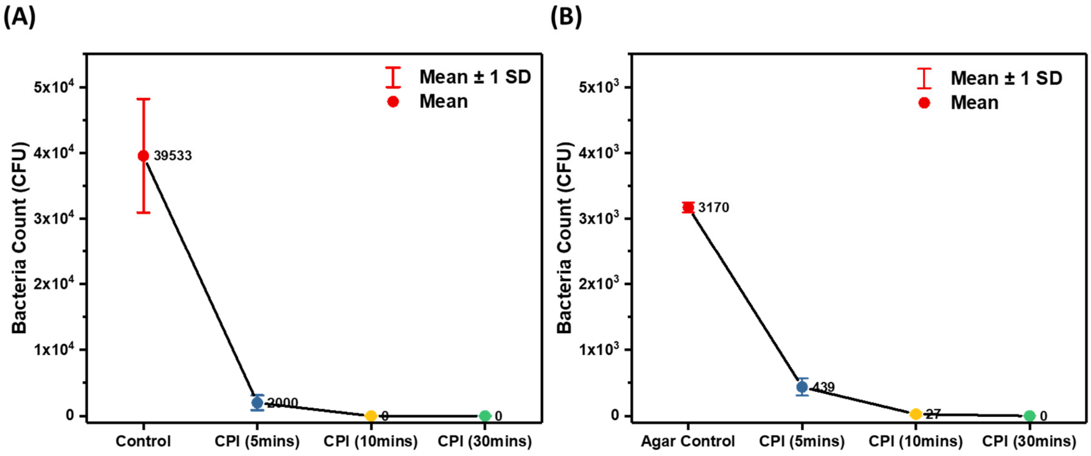

Figure S4. Bacteria count results from collected aerosolized *E. coli* (from 10^5 CFU/mL solution in the nebulizer) in the aerosol test chamber using SKC BioSampler and passive agar sampling after exposure duration to CPI based on operational timeline arrangement 2. (**a**) SKC BioSampler bacteria count results (**b**) Passive agar sampling bacteria count results.

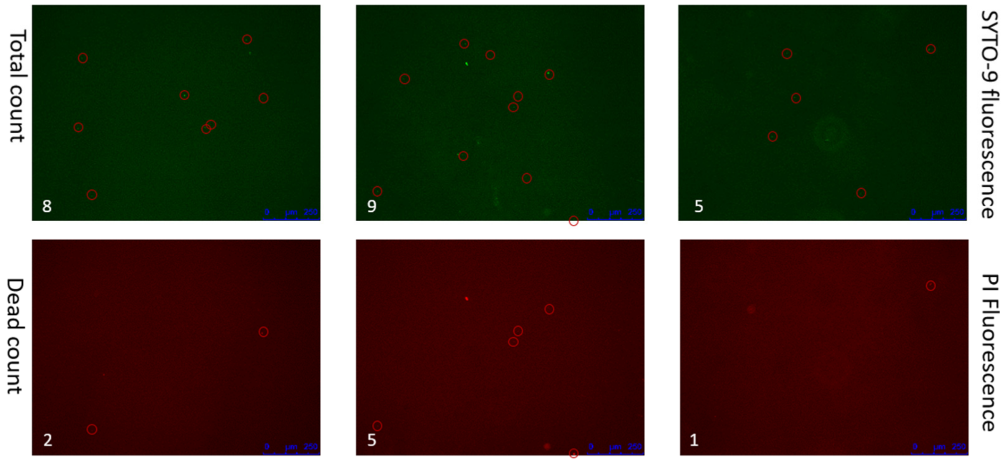

Figure S5. Fluorescence microscopy of collected SKC BioSampler samples without CPI treatment. Fluorescent bacteria in each image are circled and the count is indicated at the bottom left of each image. Few dead cells were observed stained with propidium iodide in the N21 filter setting as compared to cells stained with SYTO9 in the GFP filter setting.

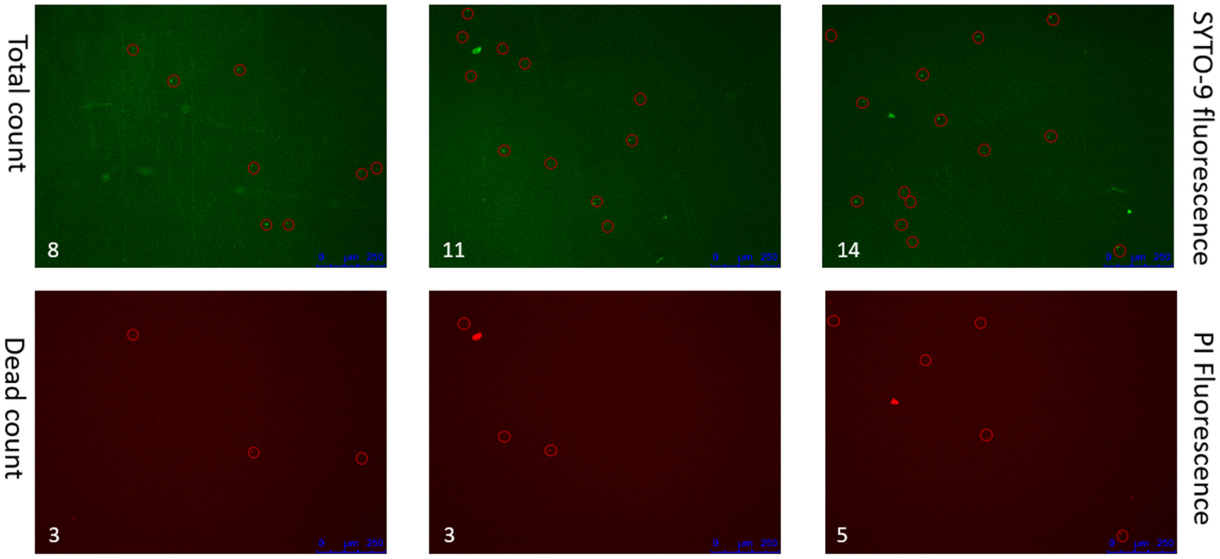

Figure S6. Fluorescence microscopy of collected SKC BioSampler samples with 5 minutes of CPI treatment under timeline arrangement 1 (CPI saturation). Fluorescent bacteria in each image are circled and the count is indicated at the bottom left of each image.

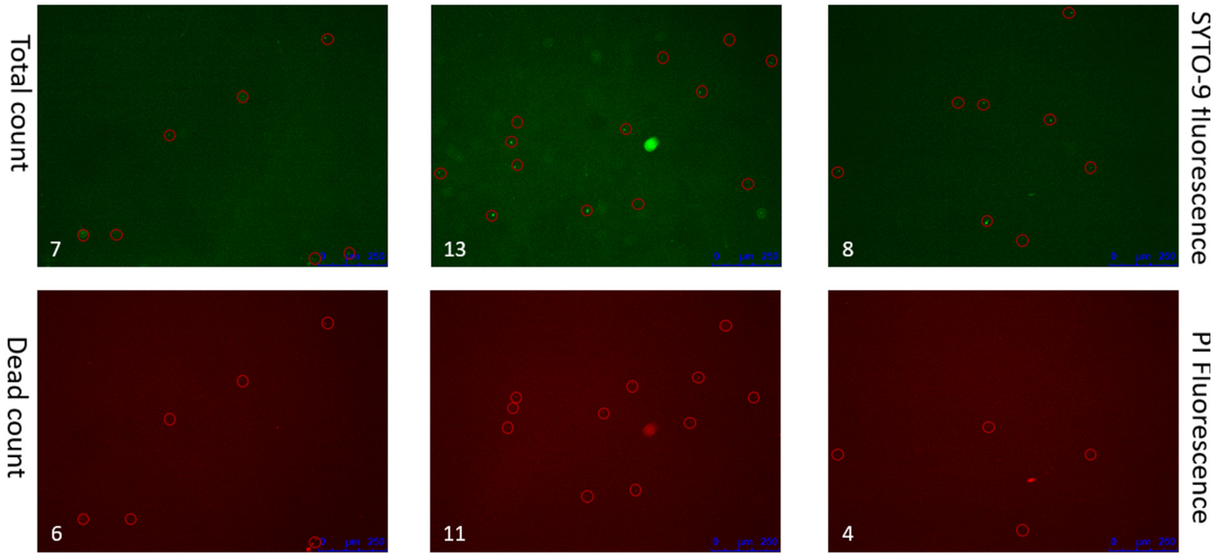

Figure S7. Fluorescence microscopy of collected SKC BioSampler samples with 15 minutes of CPI treatment under timeline arrangement 1 (CPI saturation). Fluorescent bacteria in each image are circled and the count is indicated at the bottom left of each image.

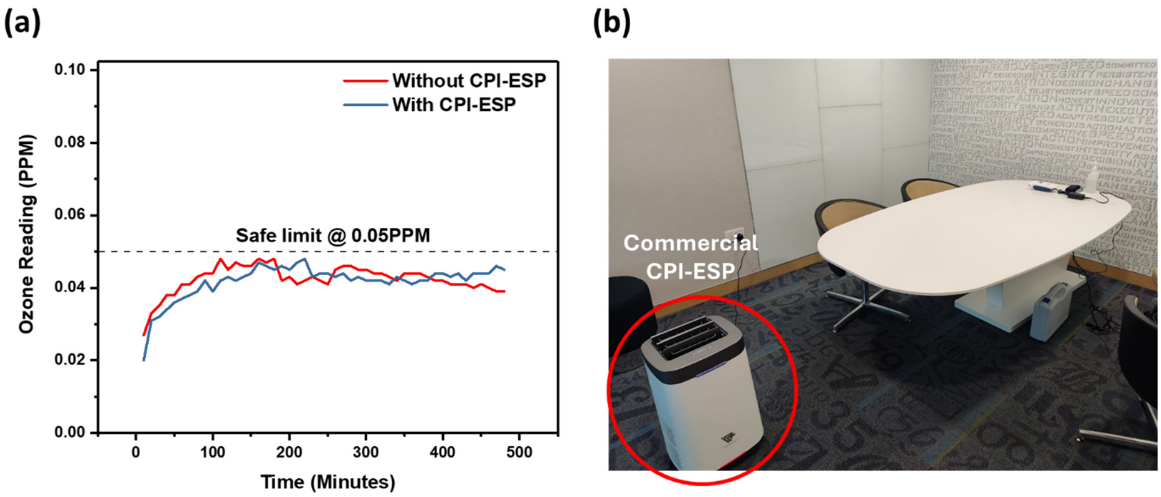

Figure S8. (**a**) Ozone monitoring data over 8 hours without and with commercial CPI-ESP equipment (**b**) Meeting room with volume space of $32.5m^3$ with the commercial CPI-ESP setup.
